# Microtubules as platforms for probing liquid–liquid phase separation in cells – application to RNA-binding proteins

**DOI:** 10.1242/jcs.214692

**Published:** 2018-06-11

**Authors:** Alexandre Maucuer, Bénédicte Desforges, Vandana Joshi, Mirela Boca, Dmitry A. Kretov, Loic Hamon, Ahmed Bouhss, Patrick A. Curmi, David Pastré

**Affiliations:** 1 SABNP Lab, Univ Evry, INSERM U1204, Université Paris-Saclay, 91025 Evry, France; 2 Institute of Protein Research, Russian Academy of Sciences, Pushchino, Moscow Region, 142290, Russia; 3 Department of Biochemistry, Boston University School of Medicine, Boston, MA 02118, USA

**Keywords:** Cellular compartment, Intrinsically disordered regions, Stress granules, Amyotrophic lateral sclerosis

## Abstract

Liquid–liquid phase separation enables compartmentalization of biomolecules in cells, notably RNA and associated proteins in the nucleus. Besides having critical functions in RNA processing, there is a major interest in deciphering the molecular mechanisms of compartmentalization orchestrated by RNA-binding proteins such as TDP-43 (also known as TARDBP) and FUS because of their link to neuron diseases. However, tools for probing compartmentalization in cells are lacking. Here, we developed a method to analyze the mixing and demixing of two different phases in a cellular context. The principle is the following: RNA-binding proteins are confined on microtubules and quantitative parameters defining their spatial segregation are measured along the microtubule network. Through this approach, we found that four mRNA-binding proteins, HuR (also known as ELAVL1), G3BP1, TDP-43 and FUS form mRNA-rich liquid-like compartments on microtubules. TDP-43 is partly miscible with FUS but immiscible with either HuR or G3BP1. We also demonstrate that mRNA is essential to capture the mixing and demixing behavior of mRNA-binding proteins in cells. Taken together, we show that microtubules can be used as platforms to understand the mechanisms underlying liquid–liquid phase separation and their deregulation in human diseases.

## Abstract (precis)

**Summary:** Confining RNA-binding proteins on microtubules allows analysis of the mixing and demixing of coexisting RNA-rich sub-compartments with liquid-like properties in living cells.

## INTRODUCTION

Multivalent interactions between RNA-binding proteins (RBPs), drive the formation of liquid-like membraneless compartments in cells (Bergeron-Sandoval et al., 2016; Castello et al., 2016; Li et al., 2012; Pak et al., 2016; Patel et al., 2015; Zhang et al., 2015). Such compartments include stress granules (Jain et al., 2016; Lin et al., 2015; Molliex et al., 2015), P granules (Strzyz, 2016), the nucleolus (Feric et al., 2016), nuclear speckles (Zhu and Brangwynne, 2015) and paraspeckles (Fox et al., 2018; Hennig et al., 2015). Through this means, RBPs are concentrated into distinct liquid phases to fulfill specific tasks related to transcription (Hnisz et al., 2017), splicing (Gueroussov et al., 2017; Ying et al., 2017), the translational response to stress, and to transport. To document this emerging field, most studies have been performed *in vitro*, but analysis of recombinant proteins is tricky for aggregation-prone RBPs such as fused in sarcoma (FUS) (Murakami et al., 2015; Patel et al., 2015) and transactive response DNA-binding protein (TDP-43; also known as TARDBP) (Conicella et al., 2016), two mRNA-binding proteins that form insoluble cytoplasmic aggregates in major neurodegenerative diseases such as amyotrophic lateral sclerosis (ALS). In addition, macromolecular crowding (Bounedjah et al., 2012) and cellular factors [RNA and protein partners (Kedersha et al., 2016), small molecules (Altmeyer et al., 2015) and post-translational modifications (Aumiller and Keating, 2016)] are difficult to mimic *in vitro* while they are potentially critical to trigger phase separation (Aguzzi and Altmeyer, 2016; Bounedjah et al., 2012). Therefore, there is a need to develop methods to probe phase separation in a cellular context (Banani et al., 2016; Patel et al., 2015; Shin et al., 2017).

**Fig. 1.**
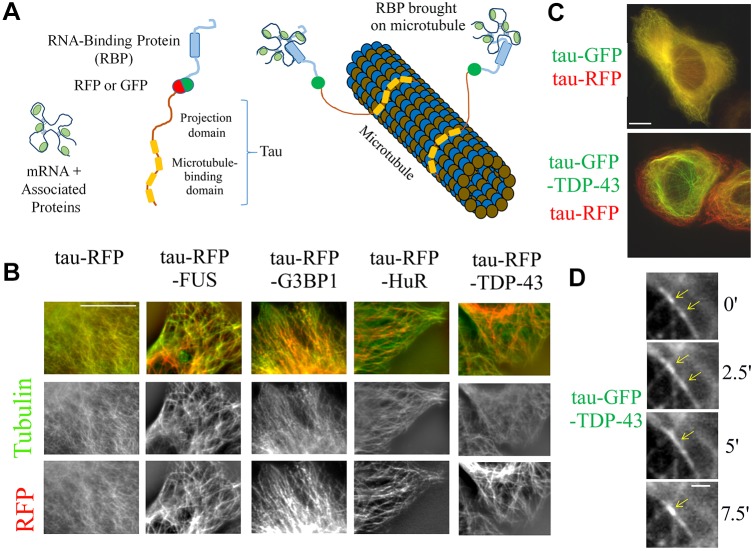
**RBPs are confined into compartments along the microtubule network after their fusion to tau.** (A) Schematic of the method used to bring RBPs on microtubules through their fusion to tau, a microtubule-associated protein. (B) All tau–RFP–RBPs tested (FUS, G3BP1, HuR and TDP-43) were brought onto microtubules in HeLa cells. tau–RFP–RBP is shown in red and anti-β-tubulin staining in green. Scale bar: 10 µm. (C) Images of Hela cells co-expressing tau–RFP and either tau–GFP and tau–GFP–TDP-43. Note the spatial segregation on microtubules induced by the fusion of TDP-43 to tau–RFP. Scale bar: 10 µm. (D) Time-lapse images (time in minutes) of tau–GFP–TDP-43. Note the fusion of two tau–GFP–TDP-43 compartments moving along a microtubule (see arrows). Scale bar: 1 µm.

Here, we present a method to probe phase separation by confining selected RBPs on microtubules in fixed or living mammalian cells (Fig. 1A,B). There are three major advantages in using this method. First, the geometry of microtubules (micrometer-long cylinders with nanometer-size diameter) enables RBPs to be confined in order to detect and quantify their spatial segregation along microtubules. Other fluorescence methods, such as fluorescence resonance energy transfer (FRET) and complementation assays (Xing et al., 2016) detect interactions between two proteins, which is inappropriate to investigate phase separation as, for example, proteins that share the same compartment may not cause a FRET signal (no direct interaction). Second, the spatial separation of virtually any protein couple can be analyzed provided that they can be brought onto microtubules and irrespective of their solubility, which is a major concern for *in vitro* investigations. Third, the compartmentalization of truncated or mutated proteins confined on microtubules can be visualized and measured, whereas protein truncation or mutation will often change the location of proteins from their original compartments, which would hinder studies on the structural basis of sub-compartmentalization in cells.

In this article, we analyzed whether four mRNA-binding proteins, TDP-43, FUS, HuR (also known as ELAVL1) and G3BP1 could form liquid phases when confined on microtubules. TDP-43 and FUS are known to form liquid droplets due to their self-attracting low complexity domains (LCDs) (Gopal et al., 2017; Murakami et al., 2015; Murray et al., 2017; Patel et al., 2015; Uversky, 2017). HuR (Aulas et al., 2015; Fialcowitz-White et al., 2007; Kedersha et al., 2016) and G3BP1 (Abrakhi et al., 2017) do not display established self-attracting LCDs. We found that confining any of these RBPs, and thus mRNAs, on microtubules leads to the formation of mRNA-rich liquid-like compartments on microtubules, irrespective of the LCD presence. To demonstrate the usefulness of our approach, the miscibility between different mRNA-rich compartments formed by bringing two different RBPs on microtubules was analyzed. We then focused our analysis on the roles of the RNA-binding domain (RBD) and the LCD in the mixing and demixing between coexisting compartments.

## RESULTS

### Confining RBPs on microtubules does not prevent their binding to mRNA and leads to the formation of RBP compartments

**Fig. 2.**
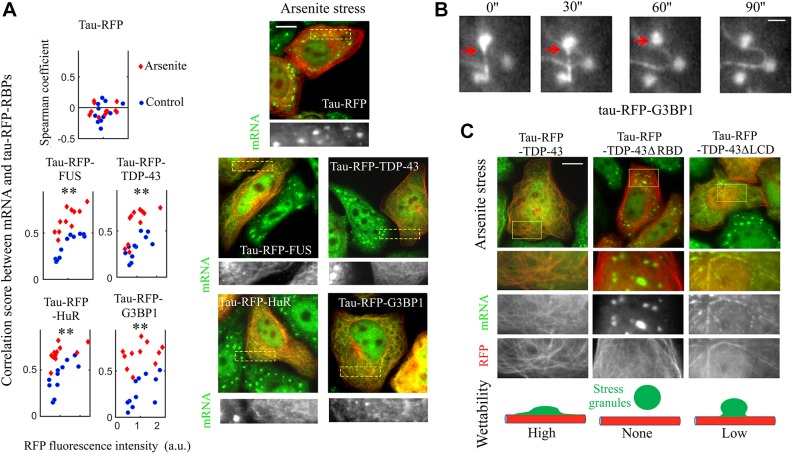
**Tau–RFP–RBPs colocalize with mRNA on microtubules and lead to the wetting of stress granules on microtubules.** (A) Right panel, spatial distribution of mRNA and tau–RFP–RBP in arsenite-treated cells (300 µM, 60 min). Arsenite leads to the formation of stress granules in control cells. Tau–RFP alone does not interact with stress granules. Fluorescent poly(T) probes were used to detect mRNA (green). Scale bar: 10 µm. Left panel: colocalization score between tau–RFP–RBPs and mRNA in control and arsenite-treated cells plotted against the tau–RFP–RBP expression levels (a.u., arbitrary units). The colocalization score correlates with tau–RFP–RBP expression levels. Arsenite further increases the colocalization score. Three independent experiments were performed, and led to the same observation. Colocalization analysis was performed as described in the Materials and Methods. Each dot represents a measurement from a single cell (*n*_cell_=11). **P<0.01 for arsenite-treated versus control cells (two-tailed *t*-test). (B) Time-lapse images (time in seconds) of tau–RFP–G3BP1 (Movie 2). Cells were treated with arsenite (300 µM) and nocodazole (500 nM) for 60 min. The red arrow indicates the interactions of a G3BP1 compartment on microtubules with stress granules. Scale bar: 2 µm. (C) Tau–RFP was fused to full-length or truncated TDP-43 in arsenite-treated cells. Either removing the LCD or RBD of TDP-43 alters the wetting of stress granules on microtubules, as summarized in the diagram in the lower panel. Scale bar: 10 µm.

To confine RBPs on microtubules, they were fused to tau (also known as MAPT) (Boca et al., 2015) (Fig. 1A,B), a microtubule-associated protein, labeled with either RFP or GFP. Tau has a higher affinity for polymerized tubulin than for free tubulin, which favors its presence on microtubules rather than in the cytosol (Lee et al., 1989). In addition, its unstructured projection domain serves as a spacer (Lee et al., 1989) to preserve RBP accessibility (Boca et al., 2015). As required in this approach, none of the RBPs investigated in the present study interact by themselves with microtubules. Through their fusion to RFP and tau, TDP-43, FUS, HuR and G3BP1 were brought onto microtubules in HeLa cells (Fig. 1B). Specific antibodies were used to confirm the presence of the proteins along the microtubule network (Fig. S1A). While endogenous TDP-43, FUS and HuR are preferentially nuclear, their fusion to tau–RFP induces a cytoplasmic location in most cells. We also noticed that all tau–RBPs are non-homogeneously distributed, which may reflect phase separation (Fig. S1B). Time-lapse imaging further reveals the presence of dynamical TDP-43 compartments that move along microtubules, and appear and disappear (Movie 1). The mobility of tau on microtubules (Janning et al., 2014; Méphon-Gaspard et al., 2016) most probably partly preserves compartment dynamics. To decipher whether RBPs account for the spatial segregation observed, we analyzed the spatial segregation of tau–RFP and either tau–GFP or tau–GFP–TDP-43 in HeLa cells co-expressing two protein fusions. Consistent with the non-cooperative binding of tau to microtubules (Butner and Kirschner, 1991), tau–RFP and tau–GFP are homogenously distributed along the microtubule network (Fig. 1B,C). On the other hand, the presence of TDP-43 generates the formation of TDP-43 compartments that are mostly located in the perinuclear region (Fig. 1C). We conclude that fusing RBPs to tau leads to their confinement on microtubules, preserves their mobility, and generates their compartmentalization on microtubules. As we want to investigate the role of mRNA in phase separation of RBPs, we also need to determine whether RBPs brought onto microtubules retain their capacity to bind to mRNA. To this end, mRNA was detected in cells by means of *in situ* hybridization with an oligo-dT probe, which recognizes the poly(A) tail of mRNA. We then measured the colocalization score between tau–RFP–RBP and mRNA and plotted this against the expression level (fluorescence intensity) of tau–RFP–RBP (Fig. 2A). The colocalization score clearly increases with tau–RFP–RBP level for all the RBPs tested. In contrast, no colocalization of mRNA was detected for tau–RFP alone whatever its expression level (Fig. 2A). Note that the basic microtubule-binding domains of tau, which may interact with acidic mRNA (Kampers et al., 1996), are not available when tau is bound to microtubules.

### Interactions of RBP compartments on microtubules with stress granules

Having shown the presence of RBP compartments, we then wondered whether RBPs located on microtubules behave like liquid phases. We observed fusion of compartments on microtubules in cells expressing tau-GFP–TDP-43, which is an indication of the liquid-like nature of these compartments (Fig. 1D). To further test this hypothesis, we aimed to probe the interactions of RBP compartments with an established mRNA-rich liquid droplet compartment, the stress granules (Bounedjah et al., 2014; Kedersha et al., 2002; Niewidok et al., 2018; Reineke et al., 2015). Stress granules are mRNA-rich cytoplasmic compartments formed after stress, here cells exposure to arsenite (300 µM for 60 min), that contain many RBPs including TDP-43, FUS, G3BP-1 and HuR (Markmiller et al., 2018; Youn et al., 2018). While we cannot exclude the presence of thin microtubule-following liquid tau–RFP compartments, tau–RFP alone does not promote the interaction between microtubules and stress granules (Fig. 2A; Fig. S3A). Free tau may be found in stress granules (Vanderweyde et al., 2016), but the availability of its microtubule-binding domains is required for its interaction with mRNA (and also for its self-attraction) (Ambadipudi et al., 2017). In contrast to tau–RFP alone, the tau–RFP–RBPs generate an enrichment of mRNA on microtubules in arsenite-treated cells (Fig. 2A; Fig. S2A), which reflects an exchange of mRNA from stress granules to RBP compartments on microtubules. In addition, while intact stress granules of round shape are present in cells expressing tau–RFP, a marked ‘wetting’ of microtubules is observed in cells expressing tau–RFP–RBPs (Fig. 2A; Fig. S2A). Such interactions even lead to the apparent disappearance of stress granules due to their spreading onto microtubules (Fig. 2A; Fig. S2A). This process is, however, reversible since the enrichment of mRNA on tau–RBP-coated microtubules correlates with the disappearance of stress granules during stress recovery (Fig. S2B). In tau–RFP–G3BP1-expressing cells treated with nocodazole at low concentration (500 nM) to partially disrupt microtubules, tau–RBPs were simultaneously found to be located in stress granules and on microtubules (Fig. 2B). We could then capture the interactions between spherical stress granules and G3BP1 compartments located on remaining microtubules via time-lapse imaging (Movie 2). The merging of G3BP1 compartments with stress granules reflects that they both have a liquid nature.

To explore the structural basis leading to the merging between RBP compartments and stress granules, we observed the interaction between stress granules and truncated TDP-43 mutants without an RBD and LCD (TDP-43ΔRBD, amino acids 267–414; TDP-43ΔLCD, amino acids 1–277). The LCD is mostly involved in multivalent protein–protein interactions, while the RBD instead mediates the binding of TDP-43 to mRNA. Deleting the RBD totally abolished the enrichment of mRNA on microtubules, and the interaction between microtubules and stress granules (Fig. 2C). By comparison, deleting the LCD hinders the wetting of microtubules by stress granules but does not completely suppress it (Fig. 2C). Hence, molecular bridges that allow the merging between TDP-43 compartment and stress granules rely mostly on the capacity of RBP to bind to mRNA and to a lesser extent on the LCD.

### Analysis of RBP distribution on microtubules allows the quantification of the level of sub-compartmentalization orchestrated by two RBPs

**Fig. 3.**
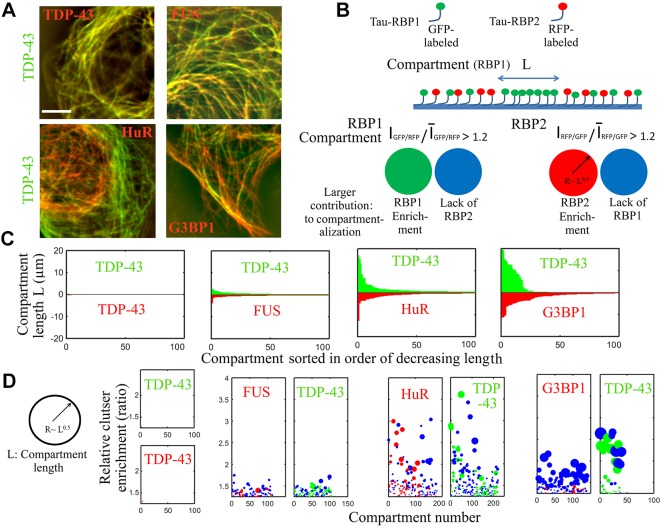
**Coexisting RBPs form distinct sub-compartments on microtubules.** (A) Images of HeLa cells co-expressing GFP- and RFP-fused tau–RBPs. Scale bar: 10 µm. (B) Schematic of the analysis of sub-compartmentalization on microtubules. Compartment length and their relative enrichment are the outputs of the described procedure (Fig. S4). *I*_RFP/GFP_ and *I*_GFP/RFP_ are fluorescence ratios. (C) Analysis of the compartment length (length analyzed along the microtubule network >0.5 mm). (D) Relative enrichment of the RBP compartments according to B.

After demonstrating the presence of mRNA-rich RBP compartments on microtubules and their merging with stress granules, we now used microtubules as nano-platforms to probe the mixing and demixing of coexisting mRNA-rich phases generated by the presence of two RBPs, labeled with GFP or RFP, on microtubules. Focusing on TDP-43, distinct micrometer-long compartments enriched in either TDP-43 or the coexisting RBP stretched along microtubules (Fig. 3A). At a larger scale, coexisting RBPs displayed a tendency to bind to either peripheral or perinuclear microtubules (Fig. S3A). In contrast, when tau–RFP and tau–GFP were fused to the same RBP, they colocalized perfectly (Fig. 3A). We also probed whether the formation of these compartments was reversible. When cells expressing tau-RBPs were placed on ice, both microtubules and tau–RBP compartments dissociated. Rewarming cells to allow microtubule reassembly restored the formation of sub-compartments (Fig. S3B).

As we found that coexisting tau–RBP compartments were spatially segregated, the microtubule nano-platform was then used to quantify the miscibility between coexisting compartments. To that end, we measured the spatial correlation of two coexisting tau–RBPs along the microtubule network (Fig. 3A,B; Fig. S4). We considered that compartments were formed whenever variations from the average RFP-to-GFP fluorescence ratio exceeded 20%. With this variation threshold, no compartment was detected between the same tau–RBP labeled with GFP and RFP. In contrast micrometer-long compartments were detected when TDP-43 and any of the other RBPs were brought onto the microtubule network (Fig. 3C). The magnitude of TDP-43 compartmentalization, however, varied with the nature of the coexisting RBPs. TDP-43 separates with G3BP1 or HuR into many micrometer-long compartments, and their relative enrichment can exceed 100% (Fig. 3D). Interestingly, TDP-43 formed poorly enriched (<50%) compartments when confined with FUS on microtubules, compared to G3BP1 and HuR. This reflects the partial miscibility of the FUS and TDP-43 phases (Fig. S3C). In agreement with this, both TDP-43 and FUS have a long self-attracting LCD and glycine-rich domains that may interact with each other. To gain insights into sub-compartmentalization mechanisms, we then reasoned that two mutually non-exclusive events contribute to RBP compartmentalization (Fig. 3B). In a given compartment, the relative enrichment of one RBP either results from its enrichment or the exclusion of the coexisting RBP (Fig. 3B). We then measured which one of these two events contributes the most to compartmentalization events measured along the microtubule network (Fig. S4D). Three proteins, TDP-43, FUS and HuR, have the capacity to accumulate into compartments (Fig. 3D, red and green spheres). Unlike TDP-43 and FUS, HuR has no apparent self-attracting LCD but its three RNA-recognition motifs (RRMs) can form multimers (Scheiba et al., 2014). In contrast, G3BP1 compartments mostly result from the absence of TDP-43 (Fig. 3D, blue spheres). G3BP1 has thus a limited ability to concentrate on microtubule segments, possibly due to the absence of LCD or other domains that may trigger self-attraction (Abrakhi et al., 2017).

### The RNA-binding domain of TDP-43 is critical for phase separation in a cellular context

**Fig. 4.**
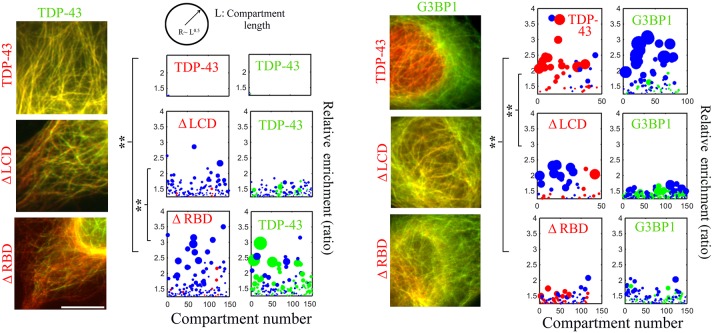
**Analysis of the role of TDP-43 domains in its compartmentalization on microtubules.** We analyzed compartmentalization of tau–GFP–TDP-43 or tau–GFP–G3BP1 (in green) coexisting with either TDP-43ΔRBD and TDP-43ΔLCD (in red) in HeLa cells. Left panel, fluorescence images. Right panel, analysis of compartmenting. Removing the RBD of TDP-43 causes demixing with full-length TDP-43 and mixing with G3BP1. Scale bar: 10 µm. **P<0.01 (two-tailed *t*-tests) for compartment enrichment data (length analyzed along the microtubule network >0.5 mm, *n*_cell_=10).

We finally took advantage of the potential of our method to investigate the mechanisms responsible for the formation of TDP-43 compartments. Notably, we explored the relative contributions of the LCD and RBD. When both tau–GFP–TDP-43 and tau–RFP–TDP-43 were brought onto microtubules, they formed a single phase on microtubules, which led to a perfect mixing between RFP- and GFP-labeled TDP-43 (Fig. 4). However, deletion of either the LCD or the RBD leads to the appearance of distinct TDP-43-rich compartments on microtubules (Fig. 4). Truncated TDP-43 most probably incorporates poorly into full-length TDP-43 compartments in which both RBD–mRNA interaction and LCD self-attraction take place. In addition, TDP-43 truncation reduces its capacity to form separated compartments on its own (Fig. 4, only blue spheres for ΔLCD and ΔRBD). We also noticed that the miscibility of TDP-43 is more affected for TDP-43ΔRBD/TDP-43 (relative enrichment ∼200%) than TDP-43ΔLCD/TDP-43 (relative enrichment >50–100%). Again, the binding of TDP-43 to mRNA appears to be critical for phase separation.

We then analyzed the consequences of protein truncation on the spatial segregation between G3BP1 and TDP-43 (Fig. 4). In contrast to full-length TDP-43, both TDP-43ΔRBD and TDP-43ΔLCD display a partial miscibility with G3BP1. RBD deletion is particularly efficient at disrupting demixing (Fig. 4). TDP-43 truncations most probably reduce the capacity of TDP-43 to become confined into compartments, leading to its partial mixing with G3BP1. Taken together, these results show that deleting the LCD or RBD can either promote or inhibit spatial segregation depending on the nature of the coexisting RBP, here TDP-43 or G3BP1.

## DISCUSSION

Phase separation of RBPs is considered to be the mechanism behind the formation of membraneless organelles in cells, including stress granules, the nucleolus, paraspeckles, nuclear speckles, Cajal bodies and others (Mitrea and Kriwacki, 2016). Besides concentrating specific biomolecules, the dynamic nature of these organelles allows the exchange of RNA and proteins with their environment, which is critical for their biological functions. However, it is necessary to be able to manipulate RBPs and analyze their respective spatial distribution in order to decipher the mechanisms underlying multilayered compartmentalization (Feric et al., 2016; West et al., 2016). Most of the recent studies are based on *in vitro* reconstitution, which often requires substantial modifications of RBPs to ensure their solubility. Therefore, there is a need to develop new approaches to probe protein phase behavior in a cellular context. To address this issue, Brangwynne and colleagues recently fused the intrinsically disordered region (here called the LCD) of different RBPs to a photolyase homology region, which is known to self-associate upon light exposure (Shin et al., 2017). Through this interesting approach, the kinetics of phase separation triggered by light exposure could be recorded for different LCDs. Here, we used microtubules as platforms to probe the mixing and demixing of RBPs in cells, irrespective of their natural location or abundance. Probing compartmentalization on microtubules also makes it possible to analyze the formation of nanometric compartments that are out of reach to conventional light microscopes. Although this approach provides otherwise unattainable information, possible biases need to be considered and probed when interpreting the results. Importantly, for each RBP fused to tau, the dynamics or reversibility of the formed sub-compartments, if any, should be checked in an effort to differentiate liquid–liquid phase separation from irreversible aggregation on microtubules (Fig. 2; Fig. S3C). Our observation that RBP-containing granules in stressed cells could ‘wet’ RBP-decorated microtubules also illustrates the liquid-like nature of the elongated compartments that we observed along the microtubule network. Finally, it should be noted that microtubule dynamics is reduced after tau expression (Lee et al., 1989), which may alter cell physiology.

**Fig. 5.**
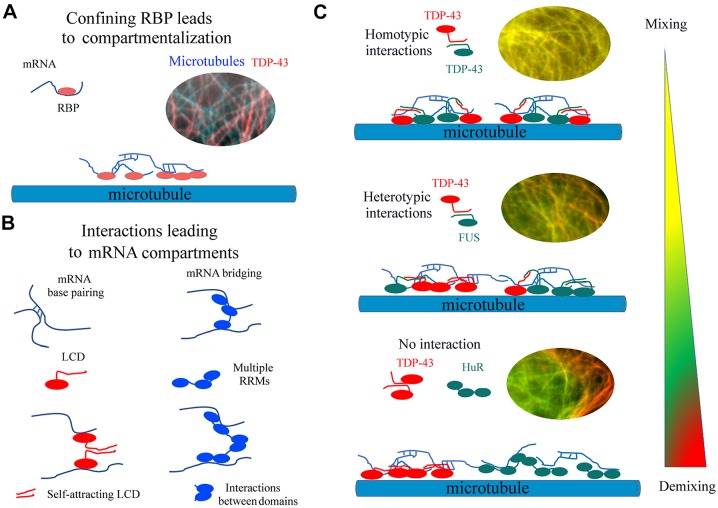
**Schematics of mechanisms behind compartmentalization of RBPs on microtubules.** A, Confining RBPs on microtubules leads to the formation of RNA-rich compartments. B, Molecular interactions accounting for the formation of mRNA granules on microtubules. RRMs, RNA-Recognition Motifs; LCD, Low Complexity Domains. C, The miscibility of liquid-like compartments depends on the interaction between coexisting RBPs.

Here, we used microtubules as platforms to investigate the behavior of four RBPs – TDP-43, FUS, HuR and G3BP1 – and TDP-43 deletion mutants. When they were fused to tau, these RBPs were brought on microtubules where they formed dynamic and reversible compartments in contrast to what was seen with tau-GFP and tau-RFP alone. They also bring mRNAs with them (Fig. 2A). Owing to the ensuing confinement of mRNAs on microtubules, RNA–RNA base-pairing probably promotes the formation of mRNA- and RBP-rich compartments (Fig. 5A; Jain and Vale, 2017; Van Treeck et al., 2018). In agreement with this model, TDP-43 compartmentalization critically depends on the presence of its two RRMs (Fig. 4), with this domain also being necessary to direct TDP-43 to stress granules (Aulas and Vande Velde, 2015). Protein–protein interactions orchestrated by LCD or other RBP domains are also important factors that modulate the relative miscibility between two coexisting compartments on microtubules. In addition to the visualization of two different sub-compartments, quantitative parameters reflecting their relative miscibility were obtained by analyzing the spatial correlation between the fluorescence signals along the microtubule filaments (Figs 3 to 5).

Among the four RBPs tested, only TDP-43 and FUS form cytoplasmic inclusions in neurons of patients affected by neurodegenerative diseases such as ALS. The presence of long LCDs with prion-like properties in both TDP-43 and FUS is most probably responsible for this. Here, we find that TDP-43-rich compartments do not mix with G3BP1- and HuR-rich compartments, most probably owing to the absence of heterotypic interaction (Fig. 5C). On the other hand, the partial mixing between TDP-43- and FUS-rich granules might reflect heterotypic interactions between the TDP-43 and FUS LCDs (Kim et al., 2010). These results therefore open perspectives to quantify partition coefficients of RBPs on a large scale in order to unravel the architectural complexity orchestrated by RBPs in cells (Nott et al., 2016). Further studies may also address the possible transition from dynamic liquid-like structures to solid-like irreversible aggregates mimicking the TDP-43-rich cytoplasmic inclusions found in patients with ALS (Li et al., 2013; Mateju et al., 2017).

## MATERIALS AND METHODS

### Preparation of plasmids and list of plasmids encoding for tau–RBPs

Vectors for mammalian expression of tau–RFP–RBPs and tau–GFP–RBPs were engineered using the gateway strategy as previously described in detail in Boca et al. (2015). Briefly, all RBPs were fused to the longest isoform of the human tau protein (accession number: NP_005901.2), which has the longest N-terminal projection domain. The human sequences of the following RBPs were inserted: TDP-43, G3BP1, HuR and FUS. The tau–RFP–TDP-43 constructs (TDP-43ΔRBD, amino acids 270–414, and TDP-43ΔLCD, amino acids 1–277) were amplified by PCR using primers containing PacI and AscI restriction sites and cloned into the ‘backbone entry plasmid’ containing the RFP–tau cassette. The two TDP-43 (1–277 or 270–414)-RFP-Tau cassettes were then transferred into the pDEST expression vector by means of the LR reaction. The accession number of RBPs and expression vectors are indicated in Table S1.

### Cell maintenance and treatments

HeLa cells were purchased from American Type Culture Collection (ATCC). Cells were maintained in DMEM containing 10% fetal bovine serum (FBS), penicillin (100 U/ml) and streptomycin (100 μg/ml). 10^6^ cells were plated in six-well plates and transfected with the indicated tau–RFP–RBP expression plasmids with Lipofectamine 2000™ reagent (Invitrogen) for 24 h after transfection. Stress granule assembly was triggered by treatment with sodium arsenite (300 µM) as indicated in the text. Cycloheximide was used at a concentration of 10 µg/ml prior to (15 min) and during arsenite treatment.

### Cell preparation for fluorescence microscopy

Fluorescence microscopy and videomicroscopy analyzes of HeLa cells expressing indicated tau–RBP constructs were performed with an inverted fluorescence motorized videomicroscope Zeiss Axiovert 200M microscope equipped with a Colibri system. Time-lapse images for videos were captured at indicated time intervals using a cooled CCD camera (Zeiss). For the preparation of fixed cells, cells were washed with PBS, fixed with ice-cold methanol for 30 min at −20°C, washed with PBS and then further fixed with 4% paraformaldehyde (PFA) in PBS for 45 min at 37°C. This double methanol/PFA fixation best reveals microtubule structures. After final washes with PBS, samples were prepared for fluorescence microscopy imaging. Anti-tubulin antibody was used to detect microtubules. See Table S2 for the references of anti-RBP antibodies used in this study. *In situ* hybridization was performed to image poly(A) mRNA in HeLa cells as follows. Cells were fixed as explained above. Cells were then incubated with 100% ice-cold methanol for 15 min at −20°C, in ice-cold 70% ethanol for 10 min at −20°C, and then 1 M Tris-HCl pH 8.0 for 5 min. A 40-nucleotide poly(T) oligonucleotide conjugated to Cy2 at 1 μg/μl in the hybridization buffer (0.005% BSA, 1 mg/ml yeast RNA, 10% dextran sulfate, 25% formamide in 2× SSC) were then used to reveal mRNAs. Slides were then placed in a humidity chamber for 1 h at 37°C with gentle shaking. Following hybridization, cells were washed twice with 4× SSC and once with 2× SSC.

To control for the reversibility of RBP compartments formed on microtubules, cells were placed on ice for 1 h in order to dissociate microtubules.

A list of reagents and resources can be found in Table S3.

### Image analysis to detect sub-compartmentalization

Fluorescence emission was collected with an oil immersion 63×1.4 NA objective. The microscope was equipped with a stable Colibri LED light (Zeiss). A high NA objective is necessary to obtain the resolution required to detect microtubules from fluorescence images. When measuring sub-compartmentalization, we selected cells displaying similar tau–GFP–RBP and tau–RFP–RBP levels for all conditions tested and for all RBPs, unless specified otherwise. The expression level was high enough to clearly distinguish the microtubule network and low enough to prevent the formation of microtubule bundles. Fluorescence analyses were performed after subtracting the background value (Subtract background tool, ImageJ). Image analysis was performed as described (Fig. S4) with the following parameters: the line thickness used to record changes of fluorescence intensities was 120 nm (4 pixels); the length analyzed along the microtubule network was longer than 0.5 mm; and a compartment was detected whenever variation of the RFP:GFP fluorescence ratio exceeded 20% (Fig. S4). The enrichment of the compartment was obtained by measuring the maximal (*I*_RBP1_/*I*_RBP2_) or (*I*_RBP2_/*I*_RBP1_) value over the length, *L*, of the considered compartment. To determine the larger contribution to RBP1 compartmenting, we consider the following Boolean tests: Log[*I*_RBP1_/mean(*I*_RBP1_)]−Log[*I*_RBP2_/mean(*I*_RBP2_)]>0 where the position I_RBP1_/I_RBP2_ value was maximal. When the Boolean value is True, RBP1 enrichment is considered as the major cause of compartmenting. When the Boolean value is False, relative RBP1 enrichment is mostly due to the absence of RBP2. A similar procedure was followed for analyzing RBP2-rich compartments. Three biological replicates were performed for each condition. Fluorescence images were then analyzed to check that compartmenting results were similar for all the replicates. In a representative replicate, the variations of fluorescence intensities were recorded in two vectors for GFP (*I*_RBP1_) and RFP (*I*_RBP2_) fluorescence, respectively.

### Measurement of the colocalization score

Spearman coefficients reflecting colocalization scores on microtubules were obtained as described previously [see fig. S6 of our previous work (Boca et al., 2015; French et al., 2008)]. Briefly, images of tau–RFP–RBP and mRNA (*in situ* hybridization) obtained from fixed HeLa cells, were first spatially filtered by using fast Fourier transform (FFT). Low spatial frequencies, corresponding to features larger than 2 µm, were discarded. Microtubule structures then appeared clearly in the images of tau-RBPs and, provided that colocalization occurred, in the images of mRNA. Images of RBPs and mRNA were selected in the cytoplasm, merged, and processed using the ‘Pearson-Spearman Correlation Colocalization’ plug-in for ImageJ. Only cells which displayed a clear microtubule network were further analyzed.

## Supplementary Material

Supplementary information

## References

[JCS214692C1] Abrakhi S., Kretov D. A., Desforges B., Dobra I., Bouhss A., Pastré D. and Hamon L. (2017). Nanoscale analysis reveals the maturation of neurodegeneration-associated protein aggregates: grown in mRNA granules then released by stress granule proteins. *ACS Nano* 11, 7189-7200. 10.1021/acsnano.7b03071 28657719

[JCS214692C2] Aguzzi A. and Altmeyer M. (2016). Phase separation: linking cellular compartmentalization to disease. *Trends Cell Biol.* 26, 547-558. 10.1016/j.tcb.2016.03.004 27051975

[JCS214692C3] Altmeyer M., Neelsen K. J., Teloni F., Pozdnyakova I., Pellegrino S., Grøfte M., Rask M.-B. D., Streicher W., Jungmichel S., Nielsen M. L. et al. (2015). Liquid demixing of intrinsically disordered proteins is seeded by poly(ADP-ribose). *Nat. Commun.* 6, 8088 10.1038/ncomms9088 26286827 PMC4560800

[JCS214692C4] Ambadipudi S., Biernat J., Riedel D., Mandelkow E. and Zweckstetter M. (2017). Liquid-liquid phase separation of the microtubule-binding repeats of the Alzheimer-related protein Tau. *Nat. Commun.* 8, 275 10.1038/s41467-017-00480-0 28819146 PMC5561136

[JCS214692C5] Aulas A. and Vande Velde C. (2015). Alterations in stress granule dynamics driven by TDP-43 and FUS: a link to pathological inclusions in ALS? *Front. Cell Neurosci.* 9, 423 10.3389/fncel.2015.00423 26557057 PMC4615823

[JCS214692C6] Aulas A., Caron G., Gkogkas C. G., Mohamed N.-V., Destroismaisons L., Sonenberg N., Leclerc N., Parker J. A. and Vande Velde C. (2015). G3BP1 promotes stress-induced RNA granule interactions to preserve polyadenylated mRNA. *J. Cell Biol.* 209, 73-84. 10.1083/jcb.201408092 25847539 PMC4395486

[JCS214692C7] Aumiller W. M. Jr. and Keating C. D. (2016). Phosphorylation-mediated RNA/peptide complex coacervation as a model for intracellular liquid organelles. *Nat. Chem.* 8, 129-137. 10.1038/nchem.2414 26791895

[JCS214692C8] Banani S. F., Rice A. M., Peeples W. B., Lin Y., Jain S., Parker R. and Rosen M. K. (2016). Compositional control of phase-separated cellular bodies. *Cell* 166, 651-663. 10.1016/j.cell.2016.06.010 27374333 PMC4967043

[JCS214692C9] Bergeron-Sandoval L.-P., Safaee N. and Michnick S. W. (2016). Mechanisms and consequences of macromolecular phase separation. *Cell* 165, 1067-1079. 10.1016/j.cell.2016.05.026 27203111

[JCS214692C10] Boca M., Kretov D. A., Desforges B., Mephon-Gaspard A., Curmi P. A. and Pastré D. (2015). Probing protein interactions in living mammalian cells on a microtubule bench. *Sci. Rep.* 5, 17304 10.1038/srep17304 26610591 PMC4661529

[JCS214692C11] Bounedjah O., Hamon L., Savarin P., Desforges B., Curmi P. A. and Pastré D. (2012). Macromolecular crowding regulates assembly of mRNA stress granules after osmotic stress: new role for compatible osmolytes. *J. Biol. Chem.* 287, 2446-2458. 10.1074/jbc.M111.292748 22147700 PMC3268405

[JCS214692C12] Bounedjah O., Desforges B., Wu T.-D., Pioche-Durieu C., Marco S., Hamon L., Curmi P. A., Guerquin-Kern J.-L., Piétrement O. and Pastré D. (2014). Free mRNA in excess upon polysome dissociation is a scaffold for protein multimerization to form stress granules. *Nucleic Acids Res.* 42, 8678-8691. 10.1093/nar/gku582 25013173 PMC4117795

[JCS214692C13] Butner K. A. and Kirschner M. W. (1991). Tau protein binds to microtubules through a flexible array of distributed weak sites. *J. Cell Biol.* 115, 717-730. 10.1083/jcb.115.3.717 1918161 PMC2289193

[JCS214692C14] Castello A., Fischer B., Frese C. K., Horos R., Alleaume A.-M., Foehr S., Curk T., Krijgsveld J. and Hentze M. W. (2016). Comprehensive identification of RNA-binding domains in human cells. *Mol. Cell* 63, 696-710. 10.1016/j.molcel.2016.06.029 27453046 PMC5003815

[JCS214692C15] Conicella A. E., Zerze G. H., Mittal J. and Fawzi N. L. (2016). ALS mutations disrupt phase separation mediated by alpha-helical structure in the TDP-43 low-complexity C-terminal domain. *Structure* 24, 1537-1549. 10.1016/j.str.2016.07.007 27545621 PMC5014597

[JCS214692C16] Feric M., Vaidya N., Harmon T. S., Mitrea D. M., Zhu L., Richardson T. M., Kriwacki R. W., Pappu R. V. and Brangwynne C. P. (2016). Coexisting liquid phases underlie nucleolar subcompartments. *Cell* 165, 1686-1697. 10.1016/j.cell.2016.04.047 27212236 PMC5127388

[JCS214692C17] Fialcowitz-White E. J., Brewer B. Y., Ballin J. D., Willis C. D., Toth E. A. and Wilson G. M. (2007). Specific protein domains mediate cooperative assembly of HuR oligomers on AU-rich mRNA-destabilizing sequences. *J. Biol. Chem.* 282, 20948-20959. 10.1074/jbc.M701751200 17517897 PMC2244793

[JCS214692C18] Fox A. H., Nakagawa S., Hirose T. and Bond C. S. (2018). Paraspeckles: where long noncoding RNA meets phase separation. *Trends Biochem. Sci.* 43, 124-135. 10.1016/j.tibs.2017.12.001 29289458

[JCS214692C19] French A. P., Mills S., Swarup R., Bennett M. J. and Pridmore T. P. (2008). Colocalization of fluorescent markers in confocal microscope images of plant cells. *Nat. Protoc.* 3, 619-628. 10.1038/nprot.2008.31 18388944

[JCS214692C20] Gopal P. P., Nirschl J. J., Klinman E. and Holzbaur E. L. F. (2017). Amyotrophic lateral sclerosis-linked mutations increase the viscosity of liquid-like TDP-43 RNP granules in neurons. *Proc. Natl. Acad. Sci. USA* 114, E2466-E2475. 10.1073/pnas.1614462114 28265061 PMC5373408

[JCS214692C21] Gueroussov S., Weatheritt R. J., O'Hanlon D., Lin Z. Y., Narula A., Gingras A. C. and Blencowe B. J. (2017). Regulatory expansion in mammals of multivalent hnRNP assemblies that globally control alternative splicing. *Cell* 170, 324-339.e23. 10.1016/j.cell.2017.06.037 28709000

[JCS214692C22] Hennig S., Kong G., Mannen T., Sadowska A., Kobelke S., Blythe A., Knott G. J., Iyer K. S., Ho D., Newcombe E. A. et al. (2015). Prion-like domains in RNA binding proteins are essential for building subnuclear paraspeckles. *J. Cell Biol.* 210, 529-539. 10.1083/jcb.201504117 26283796 PMC4539981

[JCS214692C23] Hnisz D., Shrinivas K., Young R. A., Chakraborty A. K. and Sharp P. A. (2017). A phase separation model for transcriptional control. *Cell* 169, 13-23. 10.1016/j.cell.2017.02.007 28340338 PMC5432200

[JCS214692C24] Jain A. and Vale R. D. (2017). RNA phase transitions in repeat expansion disorders. *Nature* 546, 243-247. 10.1038/nature22386 28562589 PMC5555642

[JCS214692C25] Jain S., Wheeler J. R., Walters R. W., Agrawal A., Barsic A. and Parker R. (2016). ATPase-modulated stress granules contain a diverse proteome and substructure. *Cell* 164, 487-498. 10.1016/j.cell.2015.12.038 26777405 PMC4733397

[JCS214692C26] Janning D., Igaev M., Sundermann F., Bruhmann J., Beutel O., Heinisch J. J., Bakota L., Piehler J., Junge W. and Brandt R. (2014). Single-molecule tracking of tau reveals fast kiss-and-hop interaction with microtubules in living neurons. *Mol. Biol. Cell* 25, 3541-3551. 10.1091/mbc.E14-06-1099 25165145 PMC4230615

[JCS214692C27] Kampers T., Friedhoff P., Biernat J., Mandelkow E.-M. and Mandelkow E. (1996). RNA stimulates aggregation of microtubule-associated protein tau into Alzheimer-like paired helical filaments. *FEBS Lett.* 399, 344-349. 10.1016/S0014-5793(96)01386-5 8985176

[JCS214692C28] Kedersha N., Chen S., Gilks N., Li W., Miller I. J., Stahl J. and Anderson P. (2002). Evidence that ternary complex (eIF2-GTP-tRNA(i)(Met))-deficient preinitiation complexes are core constituents of mammalian stress granules. *Mol. Biol. Cell* 13, 195-210. 10.1091/mbc.01-05-0221 11809833 PMC65082

[JCS214692C29] Kedersha N., Panas M. D., Achorn C. A., Lyons S., Tisdale S., Hickman T., Thomas M., Lieberman J., McInerney G. M., Ivanov P. et al. (2016). G3BP-Caprin1-USP10 complexes mediate stress granule condensation and associate with 40S subunits. *J. Cell Biol.* 212, 845-860. 10.1083/jcb.201508028 27022092 PMC4810302

[JCS214692C30] Kim S. H., Shanware N. P., Bowler M. J. and Tibbetts R. S. (2010). Amyotrophic lateral sclerosis-associated proteins TDP-43 and FUS/TLS function in a common biochemical complex to co-regulate HDAC6 mRNA. *J. Biol. Chem.* 285, 34097-34105. 10.1074/jbc.M110.154831 20720006 PMC2962508

[JCS214692C31] Lee G., Neve R. L. and Kosik K. S. (1989). The microtubule binding domain of tau protein. *Neuron* 2, 1615-1624. 10.1016/0896-6273(89)90050-0 2516729

[JCS214692C32] Li P., Banjade S., Cheng H.-C., Kim S., Chen B., Guo L., Llaguno M., Hollingsworth J. V., King D. S., Banani S. F. et al. (2012). Phase transitions in the assembly of multivalent signalling proteins. *Nature* 483, 336-340. 10.1038/nature10879 22398450 PMC3343696

[JCS214692C33] Li Y. R., King O. D., Shorter J. and Gitler A. D. (2013). Stress granules as crucibles of ALS pathogenesis. *J. Cell Biol.* 201, 361-372. 10.1083/jcb.201302044 23629963 PMC3639398

[JCS214692C34] Lin Y., Protter D. S. W., Rosen M. K. and Parker R. (2015). Formation and maturation of phase-separated liquid droplets by RNA-binding proteins. *Mol. Cell* 60, 208-219. 10.1016/j.molcel.2015.08.018 26412307 PMC4609299

[JCS214692C35] Markmiller S., Soltanieh S., Server K. L., Mak R., Jin W., Fang M. Y., Luo E. C., Krach F., Yang D., Sen A. et al. (2018). Context-dependent and disease-specific diversity in protein interactions within stress granules. *Cell* 172, 590-604.e13. 10.1016/j.cell.2017.12.032 29373831 PMC5969999

[JCS214692C36] Mateju D., Franzmann T. M., Patel A., Kopach A., Boczek E. E., Maharana S., Lee H. O., Carra S., Hyman A. A. and Alberti S. (2017). An aberrant phase transition of stress granules triggered by misfolded protein and prevented by chaperone function. *EMBO J.* 36, 1669-1687. 10.15252/embj.201695957 28377462 PMC5470046

[JCS214692C37] Méphon-Gaspard A., Boca M., Pioche-Durieu C., Desforges B., Burgo A., Hamon L., Piétrement O. and Pastré D. (2016). Role of tau in the spatial organization of axonal microtubules: keeping parallel microtubules evenly distributed despite macromolecular crowding. *Cell. Mol. Life Sci.* 73, 3745-3760. 10.1007/s00018-016-2216-z 27076215 PMC5002045

[JCS214692C38] Mitrea D. M. and Kriwacki R. W. (2016). Phase separation in biology; functional organization of a higher order. *Cell Commun. Signal.* 14, 1 10.1186/s12964-015-0125-7 26727894 PMC4700675

[JCS214692C39] Molliex A., Temirov J., Lee J., Coughlin M., Kanagaraj A. P., Kim H. J., Mittag T. and Taylor J. P. (2015). Phase separation by low complexity domains promotes stress granule assembly and drives pathological fibrillization. *Cell* 163, 123-133. 10.1016/j.cell.2015.09.015 26406374 PMC5149108

[JCS214692C40] Murakami T., Qamar S., Lin J. Q., Schierle G. S. K., Rees E., Miyashita A., Costa A. R., Dodd R. B., Chan F. T. S., Michel C. H. et al. (2015). ALS/FTD mutation-induced phase transition of fus liquid droplets and reversible hydrogels into irreversible hydrogels impairs RNP granule function. *Neuron* 88, 678-690. 10.1016/j.neuron.2015.10.030 26526393 PMC4660210

[JCS214692C41] Murray D. T., Kato M., Lin Y., Thurber K. R., Hung I., McKnight S. L. and Tycko R. (2017). Structure of FUS protein fibrils and its relevance to self-assembly and phase separation of low-complexity domains. *Cell* 171, 615-627.e16. 10.1016/j.cell.2017.08.048 28942918 PMC5650524

[JCS214692C42] Niewidok B., Igaev M., Pereira da Graca A., Strassner A., Lenzen C., Richter C. P., Piehler J., Kurre R. and Brandt R. (2018). Single-molecule imaging reveals dynamic biphasic partition of RNA-binding proteins in stress granules. *J. Cell Biol.* 217, 1303 10.1083/jcb.201709007 29463567 PMC5881506

[JCS214692C43] Nott T. J., Craggs T. D. and Baldwin A. J. (2016). Membraneless organelles can melt nucleic acid duplexes and act as biomolecular filters. *Nat. Chem.* 8, 569-575. 10.1038/nchem.2519 27219701

[JCS214692C44] Pak C. W., Kosno M., Holehouse A. S., Padrick S. B., Mittal A., Ali R., Yunus A. A., Liu D. R., Pappu R. V. and Rosen M. K. (2016). Sequence determinants of intracellular phase separation by complex coacervation of a disordered protein. *Mol. Cell* 63, 72-85. 10.1016/j.molcel.2016.05.042 27392146 PMC4973464

[JCS214692C45] Patel A., Lee H. O., Jawerth L., Maharana S., Jahnel M., Hein M. Y., Stoynov S., Mahamid J., Saha S., Franzmann T. M. et al. (2015). A liquid-to-solid phase transition of the ALS protein FUS accelerated by disease mutation. *Cell* 162, 1066-1077. 10.1016/j.cell.2015.07.047 26317470

[JCS214692C46] Reineke L. C., Kedersha N., Langereis M. A., van Kuppeveld F. J. M. and Lloyd R. E. (2015). Stress granules regulate double-stranded RNA-dependent protein kinase activation through a complex containing G3BP1 and Caprin1. *MBio* 6, e02486-14 10.1128/mBio.02486-14 25784705 PMC4453520

[JCS214692C47] Scheiba R. M., de Opakua A. I., Díaz-Quintana A., Cruz-Gallardo I., Martínez-Cruz L. A., Martínez-Chantar M. L., Blanco F. J. and Díaz-Moreno I. (2014). The C-terminal RNA binding motif of HuR is a multi-functional domain leading to HuR oligomerization and binding to U-rich RNA targets. *RNA Biol.* 11, 1250-1261. 10.1080/15476286.2014.996069 25584704 PMC4615805

[JCS214692C48] Shin Y., Berry J., Pannucci N., Haataja M. P., Toettcher J. E. and Brangwynne C. P. (2017). Spatiotemporal control of intracellular phase transitions using light-activated optoDroplets. *Cell* 168, 159-171.e14. 10.1016/j.cell.2016.11.054 28041848 PMC5562165

[JCS214692C49] Strzyz P. (2016). Organelle dynamics: controlling phase separation of P granules. *Nat. Rev. Mol. Cell Biol.* 18, 4 10.1038/nrm.2016.168 27991507

[JCS214692C50] Uversky V. N. (2017). Protein intrinsic disorder-based liquid-liquid phase transitions in biological systems: Complex coacervates and membrane-less organelles. *Adv. Colloid Interface Sci.* 239, 97-114. 10.1016/j.cis.2016.05.012 27291647

[JCS214692C51] Van Treeck B., Protter D. S. W., Matheny T., Khong A., Link C. D. and Parker R. (2018). RNA self-assembly contributes to stress granule formation and defining the stress granule transcriptome. *Proc. Natl. Acad. Sci. USA* 115, 2734-2739. 10.1073/pnas.1800038115 29483269 PMC5856561

[JCS214692C52] Vanderweyde T., Apicco D. J., Youmans-Kidder K., Ash P. E. A., Cook C., Lummertz da Rocha E., Jansen-West K., Frame A. A., Citro A., Leszyk J. D. et al. (2016). Interaction of tau with the RNA-binding protein TIA1 regulates tau pathophysiology and toxicity. *Cell Rep.* 15, 1455-1466. 10.1016/j.celrep.2016.04.045 27160897 PMC5325702

[JCS214692C53] West J. A., Mito M., Kurosaka S., Takumi T., Tanegashima C., Chujo T., Yanaka K., Kingston R. E., Hirose T., Bond C. et al. (2016). Structural, super-resolution microscopy analysis of paraspeckle nuclear body organization. *J. Cell Biol.* 214, 817-830. 10.1083/jcb.201601071 27646274 PMC5037409

[JCS214692C54] Xing S., Wallmeroth N., Berendzen K. W. and Grefen C. (2016). Techniques for the Analysis of Protein-Protein Interactions in Vivo. *Plant Physiol.* 171, 727-758. 10.1104/pp.16.00470 27208310 PMC4902627

[JCS214692C55] Ying Y., Wang X. J., Vuong C. K., Lin C. H., Damianov A. and Black D. L. (2017). Splicing activation by Rbfox requires self-aggregation through its tyrosine-rich domain. *Cell* 170, 312-323.e10. 10.1016/j.cell.2017.06.022 28708999 PMC5553710

[JCS214692C56] Youn J. Y., Dunham W. H., Hong S. J., Knight J. D. R., Bashkurov M., Chen G. I., Bagci H., Rathod B., MacLeod G., Eng S. W. M. et al. (2018). High-density proximity mapping reveals the subcellular organization of mRNA-associated granules and bodies. *Mol. Cell* 69, 517-532.e11. 10.1016/j.molcel.2017.12.020 29395067

[JCS214692C57] Zhang H., Elbaum-Garfinkle S., Langdon E. M., Taylor N., Occhipinti P., Bridges A. A., Brangwynne C. P. and Gladfelter A. S. (2015). RNA controls polyQ protein phase transitions. *Mol. Cell* 60, 220-230. 10.1016/j.molcel.2015.09.017 26474065 PMC5221516

[JCS214692C58] Zhu L. and Brangwynne C. P. (2015). Nuclear bodies: the emerging biophysics of nucleoplasmic phases. *Curr. Opin. Cell Biol.* 34, 23-30. 10.1016/j.ceb.2015.04.003 25942753 PMC5562147

