# Supplementary material for: Microtubules as platforms for probing liquid–liquid phase separation in cells – application to RNA-binding proteins
Source: J Cell Sci. 2018 Jun 11;131(11):jcs214692. doi: 10.1242/jcs.214692 (PMC6031325; doi:10.1242/jcs.214692)
Supplement: Supplementary information [file joces-131-214692-s1.pdf]

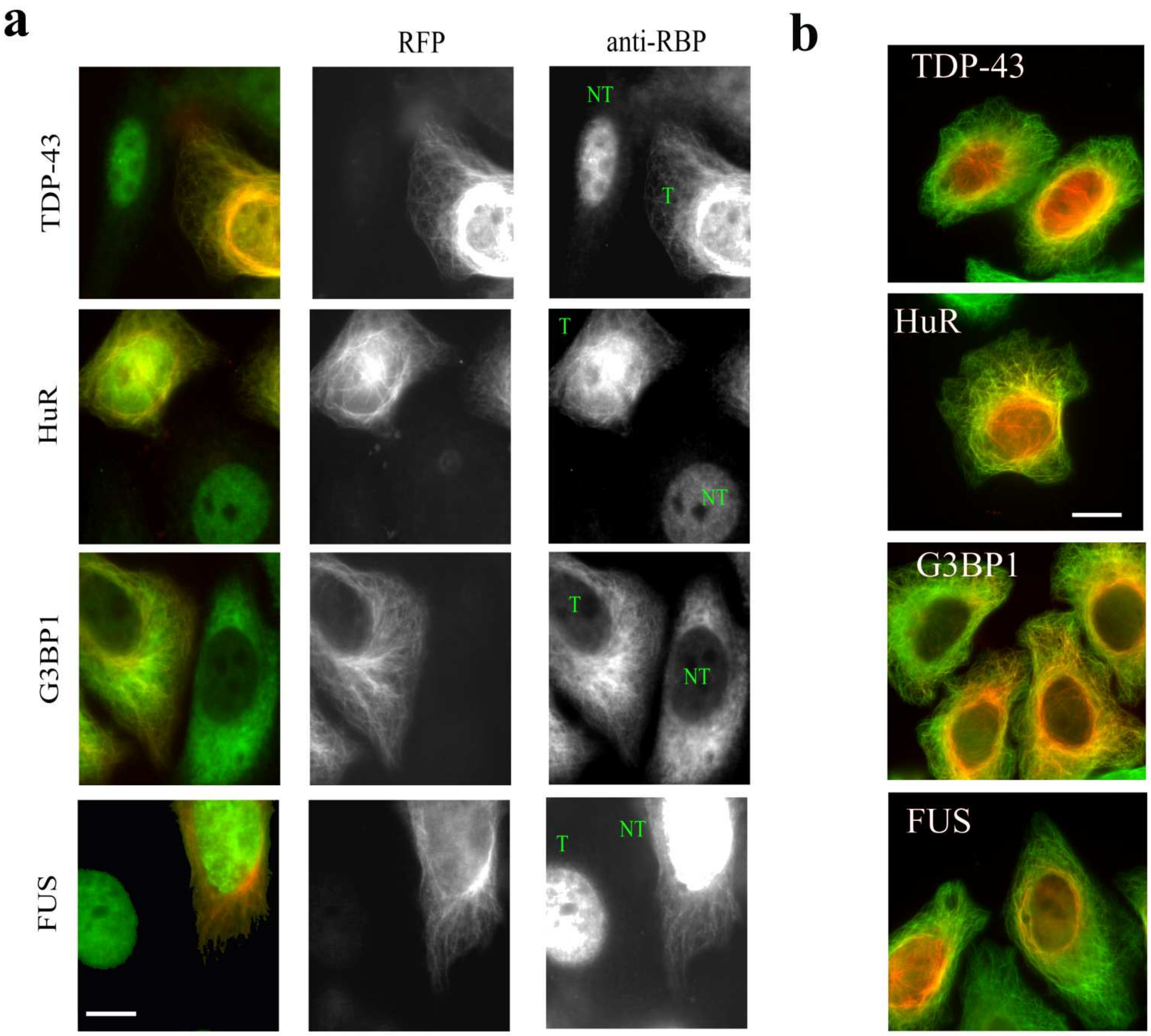

Figure S1

Supplementary figure 1: Tau-RBPs were detected on microtubules with specific anti-RBP antibodies. RBPs display a preferential perinuclear location on microtubules.

a, In HeLa cells transfected with plasmids encoding for indicated tau-RFP-RBPs, the presence of RBPS was detected by using specific anti-RBPs antibodies (see supplemental table 2) . NT: Non transfected cells expressing endogenous RBPs. T: transfected cells expressing indicated tau-RFP-RBPs. In transfected cells, we noted the appearance of microtubules win contrasts with non-transfected cells. **b**, Fluorescence images of HeLa cells expressing indicated tau-RFP-RBPs. Red (tau-RFP-RBP), Green (microtubules anti-tubulin antibody). Scale bars: 10 µm

Journal of Cell Science • Supplementary information

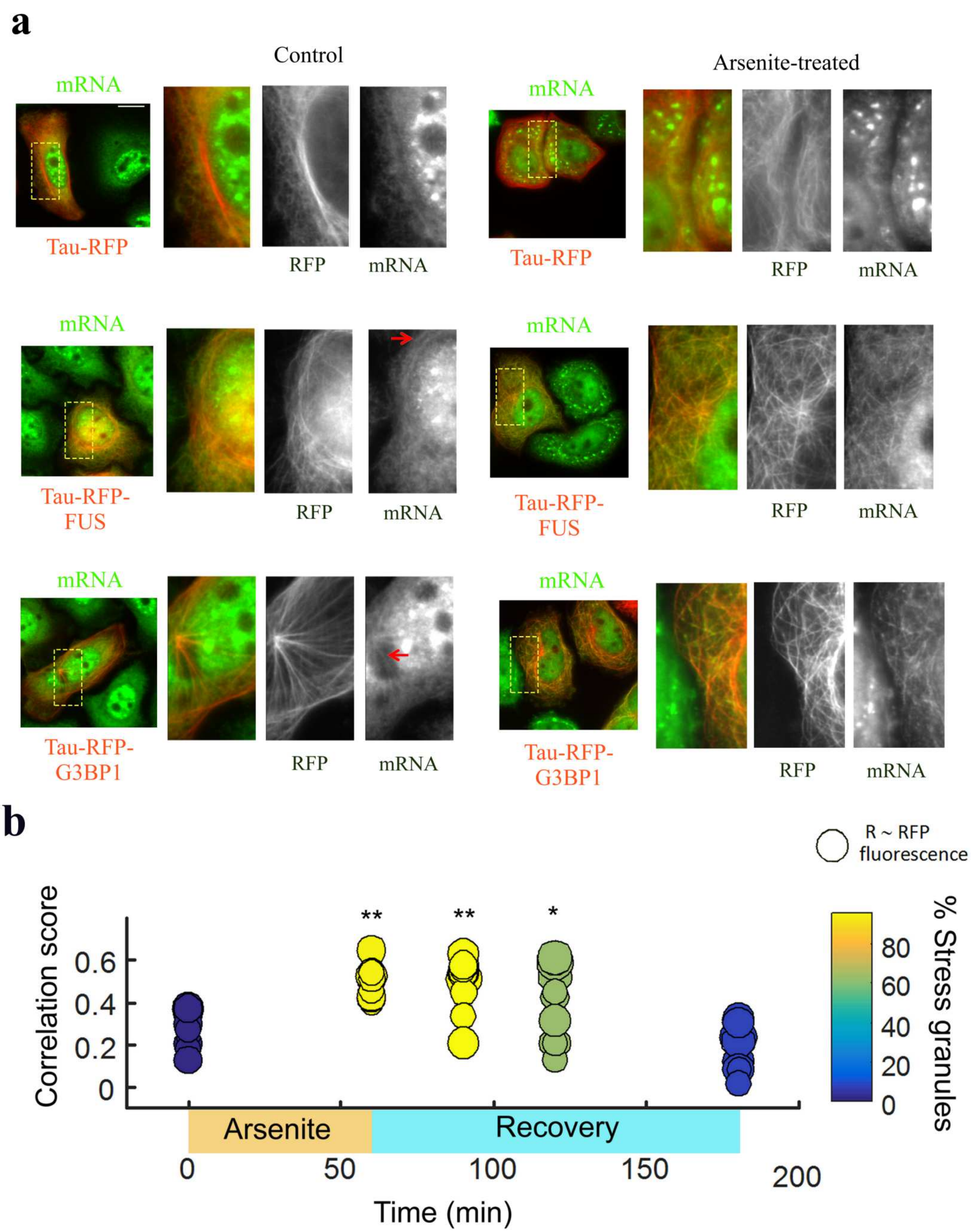

Figure S2

Journal of Cell Science • Supplementary information

Supplementary figure 2: Arsenite stress increases mRNA enrichment on microtubules of tau-RFP-RBP expressing cells.

a, Spatial distribution of indicated tau-RFP-RBP and mRNA in HeLa cells. mRNAs were detected by fluorescent poly(T) probes (in green). Tau-RFP-RBP (in red). Red arrows indicate the presence of microtubule-bound mRNA in cells expressing tau-RFP-RBP for control conditions. However, the enrichment of mRNA on microtubules is more pronounced after arenite stress compared to control. Importantly, tau-RFP-RBP does not bring mRNA on microtubules in either control or arsenite-treated cells. Scale bar: 10 µm. **b**, Spearman coefficient (correlation score) of mRNA and tau-RFP-G3BP1 fluorescence on microtubules (n=10, **P<0.01, *P<0.05, two-tailed t test versus control). Dot Radii, R, are proportional to RFP fluorescence. Arsenite was washed out to enable stress granule dissociation. Dot color represents the percentage of cells displaying stress granules among cells non-expressing tau-RFP-YB-1 (n=400). Scale bars: 10 µm

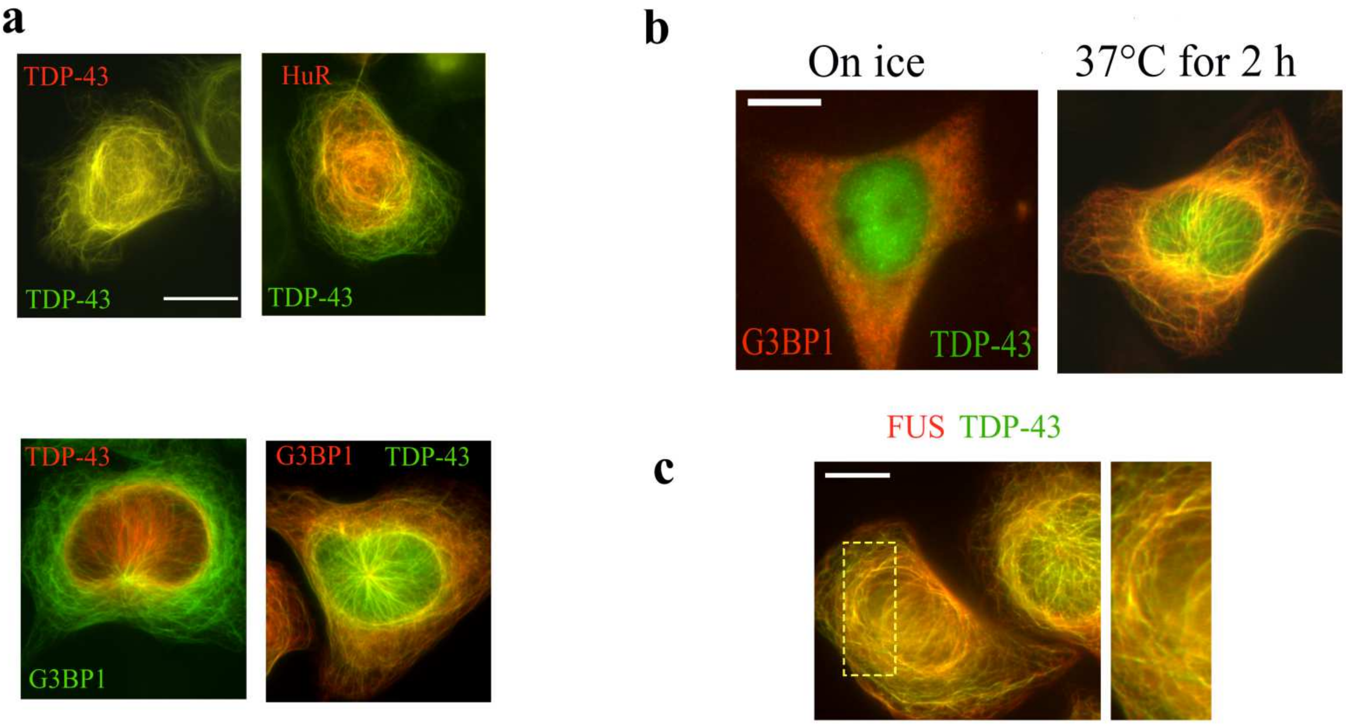

Figure S3

Supplementary figure 3: RBP couples segregate into distinct sub-compartments when coexisting on microtubules.

a, Spatial distribution of indicated GFP or RFP-fused tau-RBPs in HeLa Cells. Tau-GFP-RBP (in green). Tau-RFP-RBP (in red). Formation of distinct sub-compartments is observed for most cases, unless the same RBP was fused to tau-GFP and tau-RFP. **b,** HeLa cells expressing tau-GFP-TDP-43 and tau-RFP-G3BP1 were first placed on ice for 1 h to dissociate microtubules. Then, microtubules regrowth was triggered at 37°C for 1 h. Scale bar: 10 µm. **c**, Spatial distribution of tau-GFP-TDP-43 or tau-RFP-FUS in HeLa Cells. Note that tau-RFP-FUS and tau-GFP-TDP-43 partly share the same compartments. Scale bar: 10 µm

Journal of Cell Science • Supplementary information

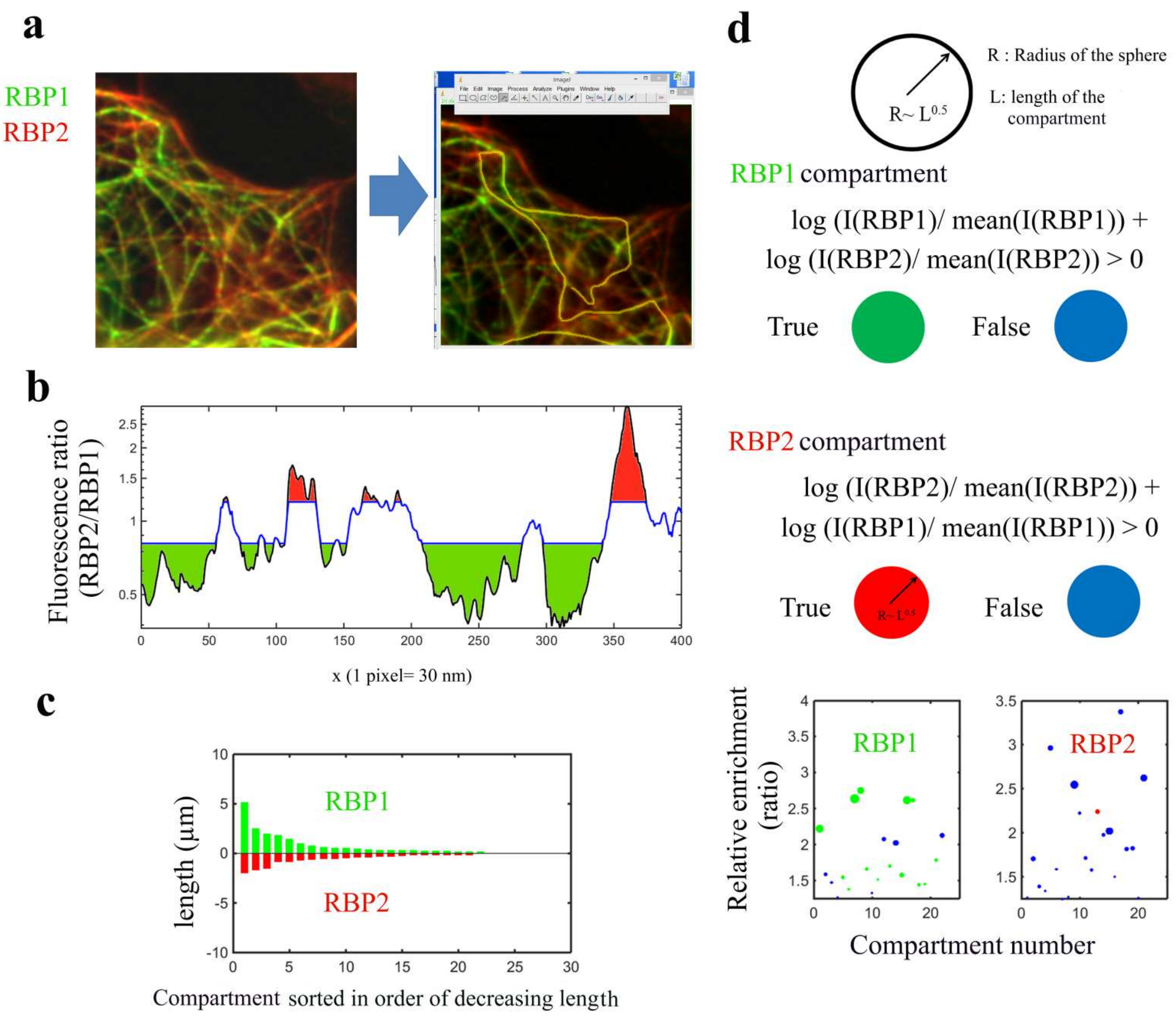

Figure S4

Supplementary figure 4: Analysis of sub-compartmentalization on microtubules.
a, Using ImageJ free hand tool, a line (yellow) along the microtubule network is drawn in cells expressing tau-GFP-RBP1 and tau-RFP-RBP2. Here RBP1 is TDP-43 and RBP2 is G3BP1. **b**, When the fluorescence RBP1/RBP2 ratio differs by more than 20% from its mean value, we consider the formation of RBP1 or RBP2 compartments. **c**, RBP1 or RBP2 compartments are sorted according to their length. **d**, Lower panel, the relative enrichment of the RBP1 and RBP2 compartment is represented. The radius of the sphere, R, is proportional to the square root of the compartment length, L. The color indicates whether compartmenting mostly results from RBP enrichment (in red for RBP1, in green for RBP2) or the depletion of the coexisting RBP (in blue).

Journal of Cell Science • Supplementary information

Movies

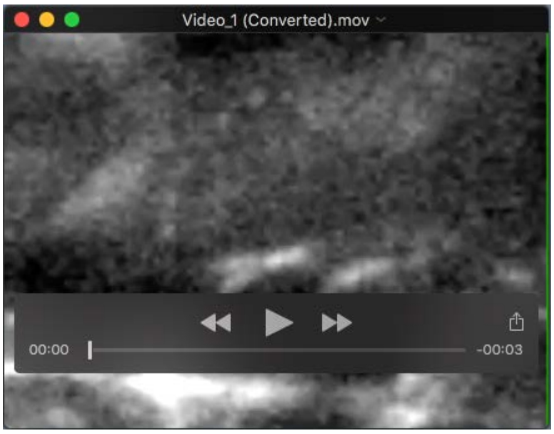

Movie 1: Tau-GFP-TDP-43 forms dynamics compartments on microtubules.
Tau-GFP-TDP-43 in Hela Cells. Time interval: 30 sec. Total duration: 15 min. Representative recordings of three independent experiments.

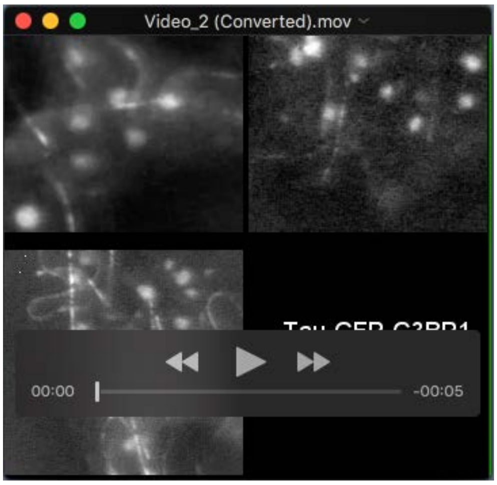

Movie 2: Stress granules interact with microtubules in Tau-GFP-G3BP1-expressing cells.
Fluorescence video-microscopy recordings of HeLa cells expressing Tau-GFP-G3BP1. Cells were treated with arsenite (300 µM) and nocodazole (500 nM) for 60 min. Time interval: 30 sec. Total duration: 7 min 30 s. Recordings of three different areas.

Journal of Cell Science • Supplementary information

SUPPLEMENTARY TABLES

Supplementary table 1: List of plasmids

Plasmids	Expression vectors	RBP accession numbers
Tau-RFP-TDP43	PEF-DEST51	NP_031401.1
Tau-RFP-FUS	PEF-DEST51	NP_004951.1
Tau-RFP-HUR	PEF-DEST51	NP_001410
Tau-RFP-G3BP1	PEF-DEST51	NP_005745.1
Tau-GFP-TDP43	PEF-DEST51	NP_031401.1
Tau-GFP-G3BP1	PEF-DEST51	NP_005745.1
GFP-HUR	pEGFP-C3	NP_001410

Supplementary tables S2 and S3: Antibodies and

Reagents Table S2 : List of primary antibodies

GENE	Antibody reference	Dilution
TDP-43	Anti-TDP-43 (Proteintech, 12892-1), produced in rabbit	1:2000
FUS	Anti-FUS (Novus, NBP2-52874), clone 190, produced in mouse	1:1000
HuR	Anti-HuR / ELAVL1 antibody (Abcam, ab54987,) produced in mouse	1:2000
G3BP1	Anti-G3BP antibody (Sigma, G6046) produced in rabbit	1:1000
Tubulin	Mouse monoclonal anti-beta-tubulin antibody (Home made, clone E7)	1:4000

Table S3: List of reagents or resources

REAGENT or RESOURCE	SOURCE		IDENTIFIER
Sodium (meta)arsenite	Sigma		S7400
Nocodazole	Sigma		M1404
Lipofectamine 2000	Thermofisher scientific		11668-019
Heat inactivated FBS	Life technologie		10500-064
DMEM+glutamax	Life technologie		31966-021
Alexa fluor 594 goat anti-mouse	Invitrogen		A11005
Alexa fluor 488 goat anti-mouse	Invitrogen		A21121
Paraformaldehyde	Alfa Aesar		43368
CELLS	SOURCE		IDENTIFIER#
HeLa	ATCC		CCL-2

Requests for reagents may be directed to, and will be fulfilled by the lead contact David Pastré.

Journal of Cell Science • Supplementary information
